# Effect of single or multi-period use of transcutaneous acupoint electrical stimulation on postoperative nausea and vomiting in patients undergoing gynecological laparoscopic surgery: a prospective randomized double-blind trial

**DOI:** 10.1186/s12906-025-04847-5

**Published:** 2025-03-20

**Authors:** Li-dan Jin, Wan Lei, Jing Xu, Li Xing, Yao-hua Shen, Su-feng Lin, Yu-fei Chen, Ting-ting He, Xi-yang Wang

**Affiliations:** 1 Department of Anesthesiology, Linping District Maternal and Children Health Care Hospital, Hangzhou, 311199 China; 2 Department of Anesthesiology, Linping Branch Women’s Hospital, Zhejiang University School of Medicine, Zhejiang University, Zhejiang, China; 3 Department of Gynecology, Linping District Maternal and Children Health Care Hospital, Hangzhou, China

**Keywords:** Acupuncture points, Transcutaneous electric acupoint stimulation, Neiguan (PC6), Hegu (L14), Gynecological laparoscopy, Postoperative nausea and vomiting

## Abstract

**Objective:**

To evaluate the efficacy of different periods for the prevention of postoperative nausea and vomiting (PONV) in patients undergoing gynecological laparoscopy with transcutaneous electrical acupoint stimulation (TEAS).

**Design:**

Prospective, randomized, double-blind trial.

**Setting:**

An academic medical canter specializing in the care of women and children.

**Population or sample:**

A total of 120 women were enrolled.

**Methods:**

Patients were randomly allocated to three groups: a single-period TEAS group (Group S, *n* = 40), a multi-period TEAS group (Group M, *n* = 40) and a non-stimulation control group (Group C, *n* = 40). TEAS was applied at specific acupuncture points preoperatively and postoperatively.

**Main outcome measures:**

The primary outcome was the incidence and severity of PONV during the 48 h after surgery and the secondary outcomes included pain, early recovery after surgery, and complication.

**Results:**

Within postoperative 0–2 h, 4–12 h, group M had less frequency and lower scores for nausea compared with group C and group S had only less frequency of nausea compared with group C at 4–12 h postoperatively. The frequency and severity of vomiting for group M were less than group S in the postoperative 2–4 h. Group M mitigated pain and reduced the rescue antiemetic compared to group S.

**Conclusion:**

Multiple-period TEAS provides greater efficacy and a longer duration of action than single-period TEAS. It effectively reduces PONV in patients undergoing gynecological laparoscopic surgery which could be a new option in multimodal prophylactic antiemetic regimes for perioperative undergoing gynecological laparoscopic surgery.

**Trial registration:**

Chinese Clinical Trials Registry, No. ChiCTR2200065802, Registered 15/11/2022. https//www.chictr.org.cn/bin/project/edit? pid=175,377.

## Introduction

Postoperative nausea and vomiting (PONV) is a common adverse reaction to anesthesia and surgery that delays recovery of gastrointestinal function and is related to a longer hospital stay and higher risks of postoperative complications [1–3]. Female patients, particularly those undergoing laparoscopic procedures, have an incidence rate as high as 80% [4], primarily due to sex-specific risk factors such as hormonal fluctuations and differences in drug metabolism [5, 6]. Additionally, laparoscopic procedures involve pneumoperitoneum and peritoneal irritation, both of which can stimulate the vagus nerve and further elevate the risk of PONV [7]. Given the high prevalence of PONV in gynecological laparoscopy, effective prevention is critical for optimizing recovery times, enhancing patient satisfaction, and reducing healthcare costs.

However, the efficacy of using single anti-emetic drugs remains suboptimal, with response rates ranging from only 20–40%, and their use is often associated with adverse effects such as headache and immunosuppression [8], making it difficult to achieve an optimal balance between efficacy and safety through drugs alone. In contrast, multiple interventions have been shown to enhance therapeutic effectiveness while minimizing these adverse effects, establishing them as a more reliable and comprehensive strategy for PONV management [9]. Nonpharmacologic interventions have gained increasing attention in recent years, with studies confirming that acupuncture, as an adjunctive therapy, can reduce the incidence of PONV by more than 20% [10], further supporting its role as a valuable component of multimodal prophylaxis [11].

As an integral component of Traditional Chinese Medicine, acupuncture has been widely used in clinical practice to prevent and alleviate nausea and vomiting. Transcutaneous electrical acupoint stimulation (TEAS) is a modern adaptation of acupuncture that delivers electrical impulses through surface electrodes to stimulate specific acupoints, thereby modulating autonomic function and neurotransmitter activity to exert antiemetic effects [12]. This technique has attracted growing interest for its potential efficacy in reducing PONV [13, 14].

However, most available studies have focused on single-period TEAS interventions, with limited exploration of the potential benefits of multiple-period TEAS, particularly in gynecologic laparoscopic surgery. Given TEAS’s proposed mechanisms of autonomic regulation and neurotransmitter modulation, we hypothesize that multiple-period TEAS may provide superior regulation compared to single-period, thereby leading to a greater reduction in PONV among laparoscopic surgery patients. Therefore, our study aims to identify a more effective TEAS strategy for PONV prevention, with the goal of reducing postoperative complications, improving perioperative quality of life, enhancing patient satisfaction, and lowering healthcare costs.

## Methods

### Study design

This prospective, randomized, double-blind trial was approved by the Research Ethics Committee of Hangzhou Linping District Maternal and Children Health Care Hospital (No. LLSC-KYKT-2022-0016-A, 09/06/2022) and registered in the Chinese Clinical Trials Registry (Registration No. ChiCTR2200065802, 15/11/2022). The study was conducted in accordance with the Declaration of Helsinki, and written informed consent was obtained from all participants. To facilitate patient enrollment and data collection, participants were offered free treatment vouchers as part of the study protocol.

### Patients

Patients who met the following criteria were considered eligible for the trial: American Society of Anesthesiologists physical status (ASA) I ~ II, aged between 18 and 65 years old, without underlying diseases, and scheduled to undergo gynecological laparoscopic surgery.

Patients meeting the following criteria were excluded from the study: neural damage or infection along the meridian at which the acupoints lay; use of antiemetic in the previous week; regular use of opioids or alcohol addicts; mental allergy or severe fear of TEAS; uncontrolled diabetes, severe coagulopathy, or cardiac, central nervous system, or psychiatric disorders; participation in other clinical trials within the last 4 weeks; recent use of TEAS or acupuncture (To minimize potential confounding factors, we excluded participants who had any treatment in the category of Traditional Chinese Medicine acupoint acupuncture in the past 4 weeks).

Drop-out criteria were as follows: operation time was more than 3 h; violation of the test program (such as failure to follow the prescribed anesthesia regimen or data collection procedures); occurrence of serious adverse events or the subjects requested to withdraw during the test.

### Randomization and blinding

Eligible patients were randomly assigned to the single-period TEAS group (Group S), the multi-period TEAS group (Group M), or the control group (Group C) in a 1:1:1 ratio using a computer-generated randomization sequence. Allocation concealment was ensured by using sealed, opaque, and sequentially numbered envelopes. A nurse, who was not involved in patient assessment was responsible for opening the envelopes and informing the TEAS technicians of the assigned group immediately before the intervention. The technicians administering the TEAS therapy and anesthesia were aware of the allocation and did not participate in outcome measurements. Postoperative follow-up observers were not involved in any treatment and were kept blinded to the allocation. All investigators in the trial adhered strictly to established procedures to maintain a clear separation between the outcome assessors and the intervention team.

### Interventions

**Fig. 1:**
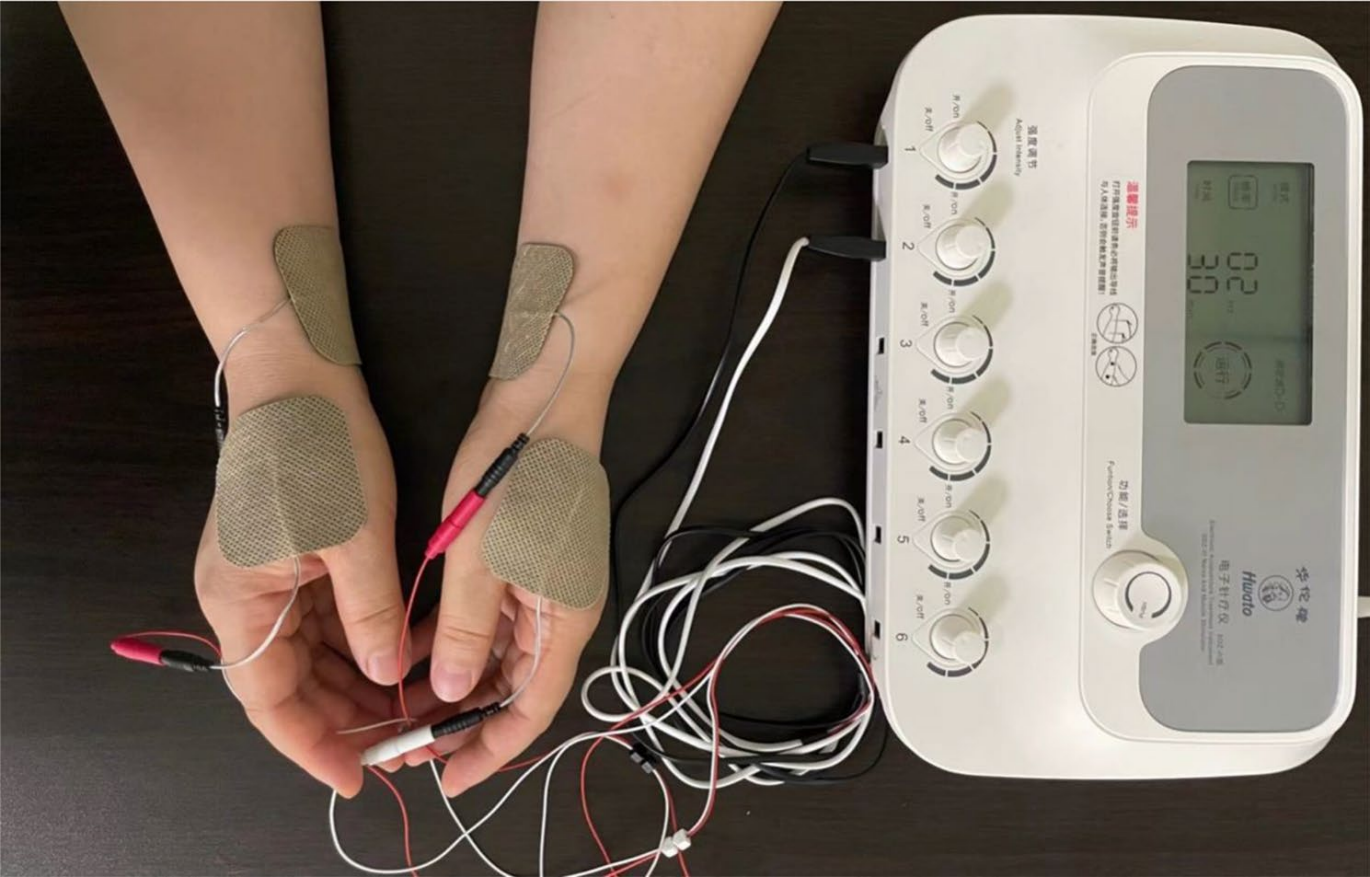
Location of acupuncture points in the trial and the connection between acupoints and the electronic acupuncture treatment instrument

All patients received general anesthesia without premedication. An experienced acupuncturist from the department of Traditional Chinese Medical performed TEAS treatments. Acupoints used in the treatment group were referenced from the China National Standard Nomenclature and Location of Meridian Points (GB 12346 − 2021) [15]. Gel electrodes were applied to the skin after it had been cleaned with ethyl alcohol. Then connected to two guide wires, which were attached to the Hwato electronic acupuncture treatment instrument (model SDZ - III, Suzhou Medical Appliances Co., Ltd., Suzhou, China), each patient required four electrodes. The selected acupoints and their locations were shown in Fig. 1. The acupoints were then stimulated electrically with an intensity adjusted to the maximum tolerance level of the patient (6–10 mA), with a dense-disperse wave, 2 and 100 Hz in frequency for 30 min in every period [16, 17]. In this study, Neiguan (PC6) and Hegu (LI4) acupoints were selected. Research has shown that PC6 is primarily used to treat hiccups, vomiting, and stomach pain, while LI4 helps strengthen the spleen and stomach, promote blood circulation, and nourish the meridians of the limbs. All patients received the same anesthesia and acupoint (PC6 and LI4) [18]. The participants in the single-period TEAS group (group S) received stimulation at acupoints, which was performed before anesthesia only and without any electrical stimulation therapy at any time. Participants in the multi-period TEAS group (group M) received stimulation treatment at three points: before anesthesia, immediately after surgery, and 2 h later. The non-stimulation control group (group C), in which patients received a sham stimulus at the acupoints by cutting off the output wires, provided no current output to the patients even though the equipment was turned on.

### Anesthesia and perioperative management

Patients were routinely fasted from solids for 6 h and liquids for 2 h before surgery. Anesthesia was administered to all subjects with propofol (2 mg/kg), midazolam (0.03 mg/kg), sufentanil (0.4 μg/kg), and tracheal intubation was facilitated by administering cisatracurium (0.2 mg/kg). Following endotracheal intubation, anesthesia was maintained with 2-3% sevoflurane (inspired concentration) and a target-controlled infusion of propofol and remifentanil. The depth of anesthesia was monitored using the bispectral index (BIS). Concentrations at the affected sites of propofol and remifentanil were adjusted to the hemodynamic index and BIS, and additional sufentanil was administered at doses deemed appropriate by the anesthesia provider. Patient’s lungs were mechanically ventilated in a volume-controlled mode with a tidal volume of 8–10 ml/kg body weight during the operation. In three groups, ondansetron 4 mg was infused 20 min before the end of the surgery, sevoflurane inhalation was discontinued, and remifentanil and propofol infusions were discontinued 10 min before the end of the surgery. Patients were extubated and transferred to the Post Anesthesia Care Unit (PACU) after surgery. All the patients ripped out the electrodes until the next day after surgery. If the score of pain (by VAS) was > 3, 0.3 g Ibuprofen (oral intake) was given. If patients experienced nausea or vomiting and the score was > 3 during the hospital stay, 4 mg ondansetron (intravenously guttae) was administered as rescue medication. All intravenous anesthetics were calculated based on the ideal body weight of each patient.

### Outcome measures

The primary outcome was the PONV score and frequency at seven period intervals (T1: 0–2 h, T2: 2–4 h, T3: 4–6 h, T4: 6–12 h, T5: 12–24 h, T6: 24–36 h, T7: 36–48 h) after the operation. The score of pain was also observed.

Patients were provided with a standardized diary to record severity and frequency of nausea using an 11-point numerical rating scale (with 0 = “no nausea” and 10 = “worst nausea imaginable”), with episodes of vomiting taken in 7- period intervals, beginning at the time of surgery for a total of 48 h. The diary also included an 11-point numerical rating scale and frequency for vomiting (with 0 = “no undesired vomiting at all” and 10 = “undesired vomiting as bad as it can be”) within the 48 h period after the operation. The visual analog score for pain (VAS: The VAS consisted of a 10-cm horizontal line anchored at one end with the words “no pain” (defined as 0 points) and at the other end with the words “worst pain imaginable” (defined as 10 points) within the 48 hours period after surgery were recorded.

The secondary outcomes included: the frequency of rescue antiemetics and analgesics; the early recovery outcomes indicators (the time of first flatus, bowel motion, and ambulation); and the length of hospital stay (the length of hospital stay is defined as the period from the end of surgery to discharge from the hospital).

The assessors on the team recorded and checked patient diary each morning following surgery to ensure that all relevant information was thoroughly collected before patient discharge.

### Statistical analysis

The sample size was evaluated by formula method ($\:n=\frac{{2\stackrel{-}{p}\stackrel{-}{q}\left({z}_{\alpha\:}+{z}_{\beta\:}\right)}^{2}}{{\left({p}_{1}-{p}_{2}\right)}^{2}}$). The primary indicator of sample size was the rate of PONV during the surgery. The preliminary experiment had 15 cases in each group; the rate of PONV in Group S and Group C was 6.6% and 40%, respectively. Therefore, a sample size of 34 in each group was determined to be required for $\:\beta\:$ value of 0.10 and $\:\alpha\:$ value of 0.05. Assuming a 15% dropout rate and considering the possibility of data loss, a sample size of at least 40 was needed in each group.

All data were tested for normally distributed using the Shapiro-Will test. Normally distributed statistical data were analyzed with One-way analysis of variance and expressed as mean ± SD. Non-normally distributed data were analyzed with the Kruskal-Wallis test and expressed as the median (Q1, Q3) or ratio. Variables measured at multiple time points were analyzed using repeated-measures analysis of variance. GraphPad Prism version 9.0 (GraphPad Software Inc., San Diego, CA, USA) and IBM SPSS Statistics for Windows version 26.0 (IBM Corp, Armonk, NY, USA) were used for data analysis. *p* < 0.05 was considered statistically significant. Missing data is minimal relatively as all data is collected during hospitalization. If any missing data remains, listwise deletion will be applied to ensure data integrity.

## Results

### Patient characteristics

**Fig. 2:**
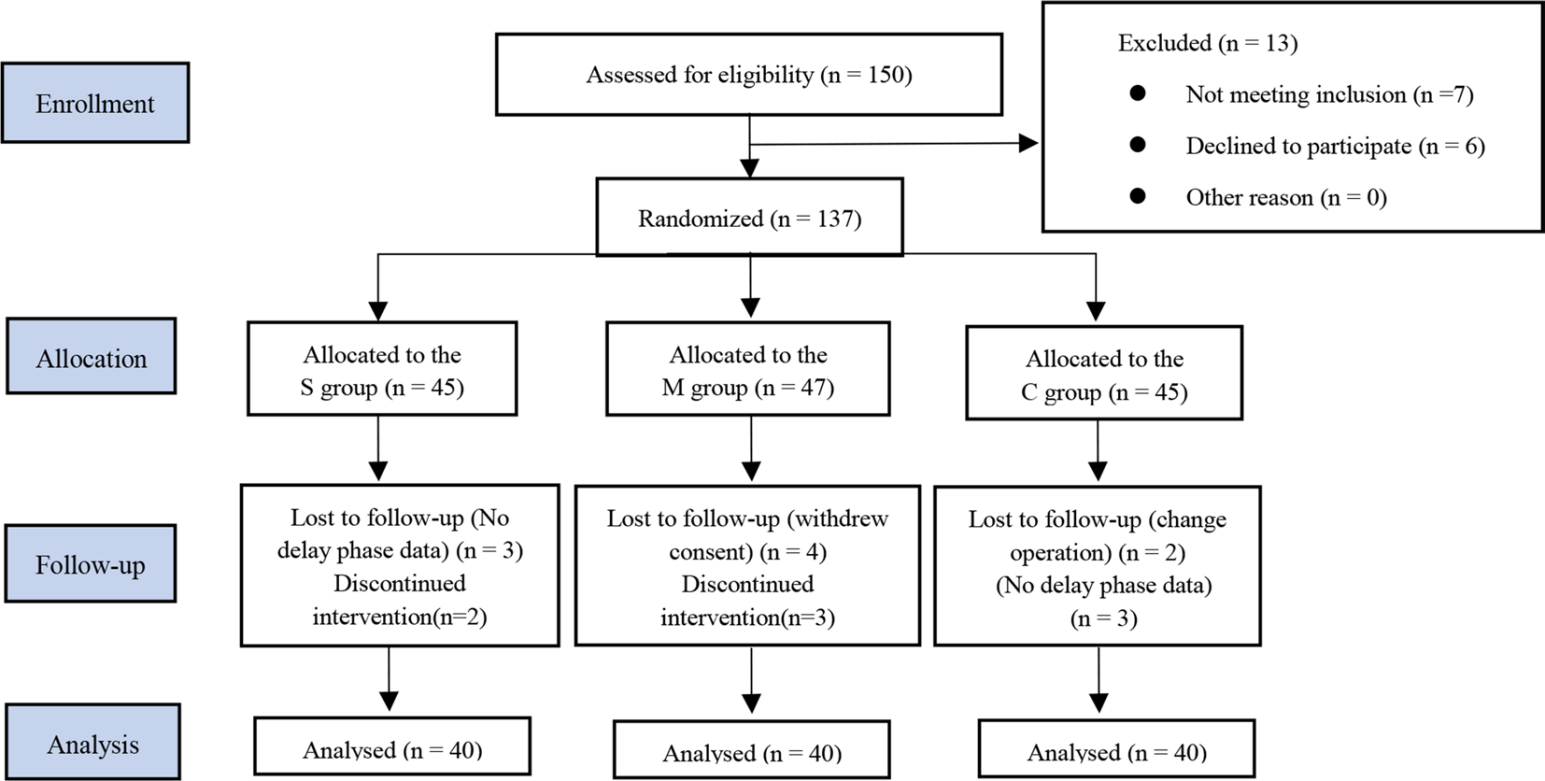
Patent flow chart

From December 12th, 2022, to June 19th, 2023, a total of 150 patients were assessed for eligibility. Thirteen patients were excluded for not meeting the inclusion criteria or declining to participate. Consequently 137 patients were stratified and randomly assigned to groups S (*n* = 45), M (*n* = 47), and C (*n* = 45). Seventeen patients discontinued participation in the trial due to various reasons: six patients lacked delay phase data, five patients did not receive the scheduled treatment, four patients withdrew due to surgery cancellation, and two patients transitioned to alternative surgical protocols. Ultimately, 120 patients completed the study and underwent analysis (Fig. 2). No significant differences were observed among the three groups in demography and intraoperative characteristics, including age, height, weight, BMI, anesthesia duration and surgery duration (*p* > 0.05) (Table 1).

**Table 1:** Demographic characteristics and duration of anesthesia and operation

Characteristic	Group S (*n* = 40)	Group M (*n* = 40)	Group C (*n* = 40)	*p* - Value
Age(years)	33.4 ± 6.6	34.8 ± 6.9	33.8 ± 6.5	0.626
Height(cm)	159.3 ± 6.1	159.1 ± 5.5	159.2 ± 4.9	0.994
Weight(kg)	56.9 ± 8.5	57.1 ± 9.4	55.6 ± 5.9	0.658
Body mass index	22.5 ± 3.2	22.5 ± 3.3	22.0 ± 2.4	0.676
Operation time(min)	62.7 ± 22.3	64.5 ± 20.5	64.2 ± 21.9	0.926
Anesthesia time(min)	81.9 ± 23.5	81.3 ± 21.1	83.9 ± 23.6	0.864

Note: Group S: single-period TEAS group; Group M: multi-period TEAS intervention group; Group C: non-stimulation control group. Data are mean ± standard deviation (SD)

### The frequency and severity of PONV

**Fig. 3:**
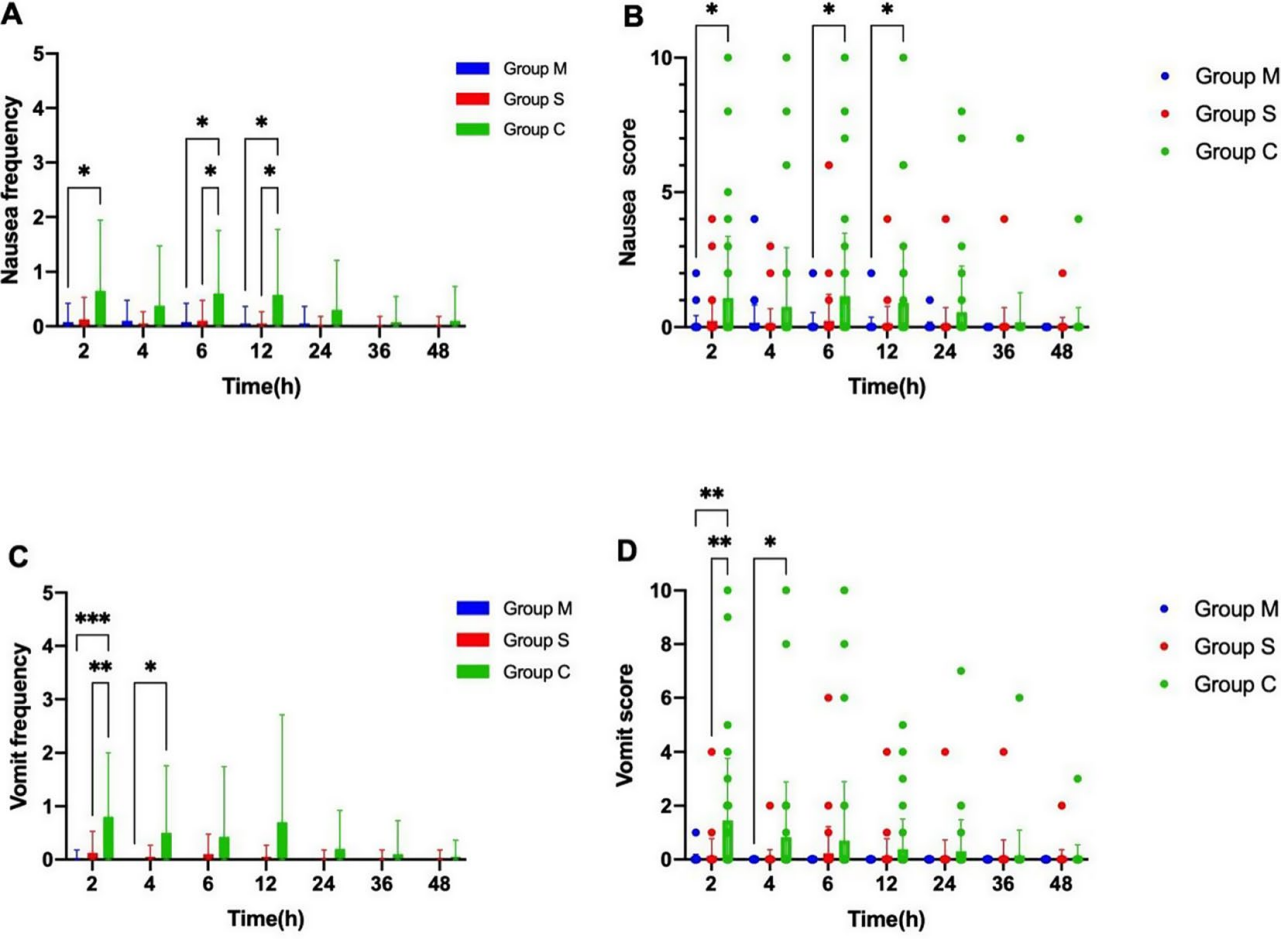
The dynamic changes in frequency and intensity of nausea and vomiting were evaluated by visual analog scale at postoperative 48 h. Note: Dynamic changes of nausea frequency(**a**), nausea score(**b**), vomit frequency(**c**) and vomit score(**d**) within 48 h after surgery. The data were expressed as mean ± standard deviation. Boxes represent mean, lines represent standard deviation. *: *p*<0.05; **p<0.01; ***p<0.0001

Overall Findings: The results revealed that PONV was observed in 20 patients (50%) in Group C, 7 patients (17.5%) in Group S, and 4 patients (10%) in Group M. The frequency and severity of PONV differed significantly among the three groups (Fig. 3).

### Frequency and severity of PONV (0–12 H)

During the first 12 h postoperatively, Groups M and S experienced less nausea and vomiting overall compared to Group C, with Group M exhibiting the lowest symptom scores.

#### Frequency and severity of nausea

Between 4 and 12 h postoperatively, both Groups M and S had a significantly lower frequency of nausea compared to Group C (*p* < 0.05). During the 0–2 h period, only Group M demonstrated a significant reduction in nausea frequency compared to Group C (*p* < 0.05) (Fig. 3A). In terms of nausea scores, Group M had significantly lower nausea scores than Group C at 0–2 h, 4–6 h, and 6–12 h postoperatively (*p* < 0.05). No significant differences were observed in Group S (Fig. 3A/B).

#### Frequency and severity of vomiting

At 0–2 h postoperatively, both Groups S and M exhibited significantly fewer and milder vomiting symptoms compared to Group C (*p* < 0.01). At 2–4 h postoperatively, Group M continued to show a significant reduction in vomiting symptoms compared to Group C (*p* < 0.05). There was no significant difference in the vomiting response among the groups beyond this period (Fig. 3C/D).

#### Frequency and severity of PONV (12–48 H)

There was no significant difference between the three groups in the number of occurrences of nausea and vomiting or the degree of scores during the first 12–48 h after surgery. (Fig. 3).

### Postoperative pain and remedies (rescue medication)

**Fig. 4:**
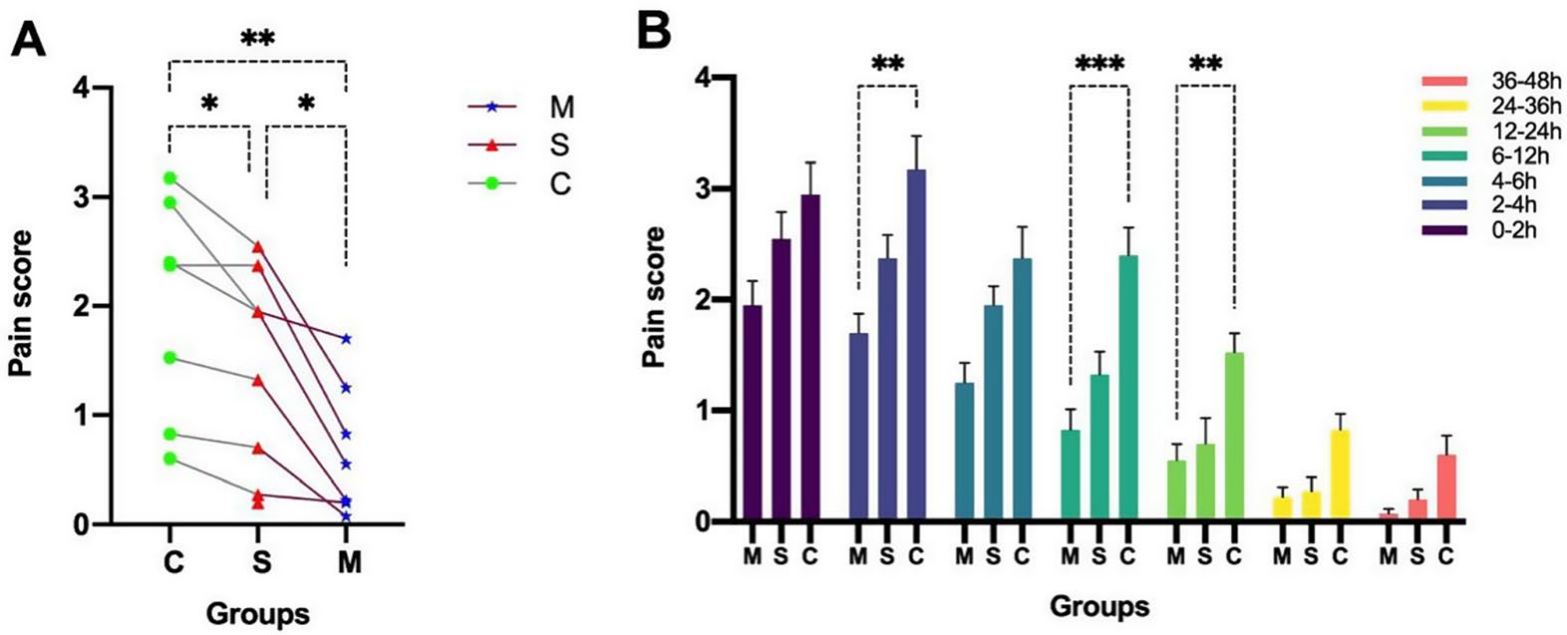
The dynamic changes of pain intensity evaluated by visual analog scale at postoperative 48 h. Note: Distribution of total dynamic changes in pain scores over seven postoperative periods (**a**) and the distribution of changes in pain scores at each postoperative time period (**b**). The data were expressed as mean ± standard deviation. Points (a) and boxes (b) represent mean, lines (b) represent standard deviation. *: *p* < 0.05; **p < 0.01; ***p < 0.0001

Groups M and S mitigated pain compared with group C (*p* < 0.05), simultaneously, the analgesic advantage of group M was more obvious than that of group S (*p* < 0.05). These advantages are mainly reflected within 2–4 h, 6–12 h and 12–24 h after surgery (as shown in Fig. 4). The incidence of rescue medication for nausea and vomiting within the first 12 h after surgery was significantly lower than in group M than in group C (*p* < 0.05). There was no significant difference in rescue medication for nausea and vomiting between groups S and C. In terms of rescue medication for postoperative pain, the difference was not significant among the three groups (Table 2).

**Table 2:** The incidence of rescue medication (case [%])

	Group S (*n* = 40)	Group M (*n* = 40)	Group C (*n* = 40)
Nausea			
0–12 h	3(7.5%)	1(2.5%) *	7(17.5%)
12–24 h	1(2.5%)	0(0%)	2(5%)
24–48 h	1(2.5%)	0(0%)	1(2.5%)
Vomiting			
0–12 h	3(7.5%)	0(0%) *	7(17.5%)
12–24 h	1(2.5%)	0(0%)	1(2.5%)
24–48 h	1(2.5%)	0(0%)	1(2.5%)
Pain			
0–12 h	7(17.5%)	8(20%)	14(35%)
12–24 h	4(10%)	1(2.5%)	2(5%)
24–48 h	0(0%)	0(0%)	1(2.5%)

Note: Group S: single-period TEAS group; Group M: multi-period TEAS intervention group; Group C: non-stimulation control group. * *p* < 0.05, *compared with the Group C*

### Comparison of other secondary outcomes among the 3 groups

The results showed that both groups M and S, which received the TEAS intervention, were significantly shorter than group C (*p* < 0.05) in the time to the first bowel motion. However, no significant differences were determined to exist in the time to first flatus, first ambulation after surgery, or hospital stay in all three groups (Table 3). No side effects related to TEAS treatment were observed during the postoperative 48 h.

**Table 3:** Comparison of other secondary outcomes among the 3 groups

	Group S (*n* = 40)	Group M (*n* = 40)	Group C (*n* = 40)	*p* - Value
First flatus(h)	18 (13, 21)	16 (10, 20)	20(13, 29)	0.061
Frist bowel motion(h)	40(34, 46)	43(31, 48)	46(41, 54)	0.03 *
First ambulate(h)	15(7,18)	8(7, 17)	10(7, 17)	0.254
Length of hospital stay(h)	91(84, 94)	93(76, 95)	93(89,96)	0.374

Note: Group S: single-period TEAS group; Group M: multi-period TEAS intervention group; Group C: non-stimulation control group. * *p* < 0.05, *compared with the Group C*

## Discussion

### Main findings

In this randomized, patient- and anesthesiologist-assessors-blind clinical trial, multiple-period TEAS significantly reduced the incidence of PONV during the first 12 h postoperatively (7.5% lower compared to single-period TEAS and 40% lower compared to control group). Additionally, it alleviated postoperative pain and expedited the first bowel movement. These findings align with our initial hypothesis and provide new evidence supporting the efficacy of multiple-period TEAS in perioperative management. To the best of our knowledge, this is the first study to apply bilateral PC6 and L14 acupoints for TEAS in patients undergoing gynecologic laparoscopic surgery while evaluating nausea and vomiting at different postoperative time points within 48 h.

Patients undergoing gynecologic laparoscopic surgery are at high risk for PONV due to multiple contributing factors, including female sex, opioid use, laparoscopic surgical stimulation, and patient positioning [1, 7]. The pathogenesis of opioid-induced PONV is thought to involve direct stimulation of vestibular apparatus and chemoreceptor trigger zone (CTZ) by opioids and decline of gastrointestinal motility [19]. Besides, laparoscopic surgery exacerbates PONV risk through diverse mechanisms, including the release of histamine, serotonin, and neurokinin-1 in response to surgical stimuli [20]. Additionally, elevated intracranial pressure and carbon dioxide levels due to the Trendelenburg position and pneumoperitoneum lead to cerebellar oedema-related ischemia and impaired oxygen metabolism associated with the Vestibular system, which further increases the risk of PONV [21].

TEAS is believed to exert its antiemetic effects through multiple physiological pathways [22]. Firstly, acupuncture stimulation promotes the release of endogenous opioids from immune cells, thereby inhibiting nociceptive signaling at peripheral nerve terminals [23]. Secondly, the main effect of TEAS in supraspinal structures stimulates neuronal pathways from the soma splanchnic neurons to the paraventricular nucleus of the hypothalamus also reduces the secretion of 5-hydroxytryptamine(5-HT) in the duodenum and suppresses the activation of the nucleus tractus solitarius in the brain-stem [24, 25]. Moreover, TEAS inhibits the sympathetic nerve and stimulates the parasympathetic nerve and hence increasing the activity of acetylcholinesterase and stimulating receptors such as nitric oxide(NO), opioid receptors, and others and regulates neurotransmitters such as 5-HT, gamma-aminobutyric acid(GABA), and catecholamines [12, 26–28]. Therefore, we hypothesize that TEAS modulates the autonomic nervous system by inhibiting sympathetic activity and enhancing parasympathetic function, which in turn increases acetylcholinesterase activity and modulates key neurotransmitters, such as 5-HT, GABA and catecholamines. Based on these mechanisms, we hypothesize that TEAS exerts its antiemetic effects by restoring autonomic balance, suppressing sympathetic overactivity, and enhancing parasympathetic function. These combined effects may contribute to reducing PONV incidence, alleviating postoperative pain, and promoting gastrointestinal recovery. Our findings are consistent with previous research demonstrating that TEAS effectively reduces postoperative nausea and vomiting, mitigates pain, and facilitates gastrointestinal function recovery [18, 29–32].

The timing and duration of TEAS application are critical factors influencing its efficacy, yet an optimal intervention strategy remains controversial [33, 34]. Our study aimed to compare the effects of single-period and multiple-period TEAS on the incidence and severity of PONV. The results demonstrated that multiple-period TEAS reduced the risk of PONV by 7.5% (M group: 10%, S group: 17.5%) compared to single-period TEAS. This finding is consistent with previous research, which indicates that perioperative stimulation is more effective in preventing PONV than preoperative stimulation alone [35]. These findings suggest that prolonged TEAS administration enhances its prophylactic efficacy against PONV [36]. According to the principles of the Traditional Chinese Medicine, the localization of acupuncture stimulation is determined by the patient’s ability to perceive Qi to ensure therapeutic accuracy [37]. Given that comprehensive perioperative management is not well tolerated by most patients, we implemented two distinct treatment protocols in a fully awake preoperative state, and the multi-period TEAS added two supplementary 30-minute treatments after surgery [17, 18]. Although our findings suggest that extending TEAS beyond a single-period enhances its antiemetic efficacy, further experimental studies are necessary to establish the optimal timing and duration of TEAS intervention.

Unlike some previous studies, our findings did not show a significant reduction in hospital stay. Li et al.’ reported that perioperative multi-acupoint TEAS with increased postoperative stimulation frequency shortened hospital stay by 12.8% compared to controls [17]. This discrepancy may be attributed to the additional acupoints used (LI4, PC6, ST36, and ST37) and the increased frequency of postoperative stimulation. Another study found that repeated and prolonged TEAS therapy during and after gastrointestinal surgery resulted in a 30% reduction in hospital stay [12]. Apart from differences in surgical types, the prolonged duration of TEAS stimulation in those studies may be the major reason for the difference in our investigation. Thus, while our findings did not show a reduction in hospital stay, they reinforce the potential benefits of TEAS in postoperative recovery, particularly for gastrointestinal function.

Importantly, no acupoint-related adverse events were observed, further supporting the safety and tolerability of TEAS regardless of the acupoints used. Additionally, compared to previous research [13, 35], our study is the first to investigate PONV incidence at different postoperative time points within 48 h for both single-period and multiple-period TEAS, filling a gap in research regarding treatment timing and duration. These findings validate the enhanced effectiveness of prolonged TEAS application and underscore its potential as a component of multimodal analgesia and PONV prevention. Given the growing emphasis on multimodal perioperative care, we recommend incorporating multiple-period TEAS into the standardized PONV prophylaxis protocols. Further training and education should be provided to optimize TEAS implementation and enhance perioperative patient comfort.

### Strengths and limitations

This study has several limitations. Firstly, as a single-center study, external validity may be limited. Although the inclusion of 30 surgeons, 20 anesthesiologists, and a sufficient number of participants introduces a degree of heterogeneity, partially mitigating this concern. Additionally, genetic predisposition, cultural perceptions of acupuncture, and variations in regional healthcare practices may influence the efficacy of TEAS. Therefore, future multicenter studies are necessary to validate our findings and assess the broader applicability of TEAS across diverse populations. Second, this study focused on a high-risk PONV population, where the therapeutic benefits of TEAS may be more pronounced. Future studies should explore TEAS efficacy in lower-risk populations to determine its broader clinical significance. Third, despite our study optimizing the perioperative TEAS protocol to balance efficacy and patient adherence, practical challenges remain in widespread implementation. Additional treatment periods and associated healthcare costs may limit accessibility in certain regions. Future research should investigate the optimal timing and duration of TEAS, balancing treatment effectiveness with clinical feasibility and resource availability.

## Conclusion

Multiple-period TEAS demonstrates superior efficacy and prolonged duration of action compared to single-period TEAS. It effectively reduces PONV, alleviates postoperative pain, and accelerates the post-surgery bowel recovery time in patients undergoing gynecological laparoscopic procedures, which presents a potential new option for multimodal prophylactic antiemetic regimens during the perioperative period for gynecological laparoscopic surgery.

## Data Availability

The datasets generated and/or analysis during the current study are not publicly available to ensure higher levels of data safety and protection, but are available from the corresponding author on reasonable request.
